# Light Intensity- and Spectrum-Dependent Redox Regulation of Plant Metabolism

**DOI:** 10.3390/antiox11071311

**Published:** 2022-06-30

**Authors:** Péter Borbély, Anna Gasperl, Tamás Pálmai, Mohamed Ahres, Muhammad Ahsan Asghar, Gábor Galiba, Maria Müller, Gábor Kocsy

**Affiliations:** 1 Agricultural Institute, Centre for Agricultural Research, ELKH, 2462 Martonvásár, Hungary; borbely.peter@atk.hu (P.B.); palmai.tamas@atk.hu (T.P.); mohamed.ahres@atk.hu (M.A.); muhammad.ahsan@atk.hu (M.A.A.); galiba.gabor@atk.hu (G.G.); 2 Institute of Biology, Department of Plant Sciences, Stress- and Cellbiology of Plants, University of Graz, 8010 Graz, Austria; anna.gasperl@uni-graz.at (A.G.); maria.mueller@uni-graz.at (M.M.); 3 Department of Environmental Sustainability, Hungarian University of Agriculture and Life Sciences, Georgikon Campus, 8360, Keszthely, Hungary

**Keywords:** antioxidants, blue light, carbon assimilation, elongated hypocotyl5, far-red light, light intensity, lipid metabolism, nitrogen assimilation, photoreceptors, reactive oxygen species, red light, secondary metabolism, sulphur assimilation

## Abstract

Both light intensity and spectrum (280–800 nm) affect photosynthesis and, consequently, the formation of reactive oxygen species (ROS) during photosynthetic electron transport. ROS, together with antioxidants, determine the redox environment in tissues and cells, which in turn has a major role in the adjustment of metabolism to changes in environmental conditions. This process is very important since there are great spatial (latitude, altitude) and temporal (daily, seasonal) changes in light conditions which are accompanied by fluctuations in temperature, water supply, and biotic stresses. The blue and red spectral regimens are decisive in the regulation of metabolism because of the absorption maximums of chlorophylls and the sensitivity of photoreceptors. Based on recent publications, photoreceptor-controlled transcription factors such as ELONGATED HYPOCOTYL5 (HY5) and changes in the cellular redox environment may have a major role in the coordinated fine-tuning of metabolic processes during changes in light conditions. This review gives an overview of the current knowledge of the light-associated redox control of basic metabolic pathways (carbon, nitrogen, amino acid, sulphur, lipid, and nucleic acid metabolism), secondary metabolism (terpenoids, flavonoids, and alkaloids), and related molecular mechanisms. Light condition-related reprogramming of metabolism is the basis for proper growth and development of plants; therefore, its better understanding can contribute to more efficient crop production in the future.

## 1. Introduction

Both spatial and temporal changes occur in the intensity and spectrum of the biologically active light radiation range (280–800 nm). Increasing altitude and latitude are associated with higher ratios of blue light (BL), photosynthetically active radiation (PAR), and ultraviolet-B (UV-B) light, whereas lower red: far-red (R:FR) ratios occur at higher latitude and during sunset. Seasonal alterations also affect the spectral composition of sunlight [1,2,3]. The R:FR ratio is constant during daylight and independent of cloud cover but is much reduced during twilight when solar elevation is less than 10° [4]. Moving north from the equator, this phenomenon becomes more pronounced and characteristic of temperate and boreal climates in the northern hemisphere. These alterations in light conditions greatly affect the metabolism and subsequently the growth and development of plants. Thus, a reduced R:FR ratio during the twilight hours, especially in the evening, has been shown to control the cessation of internode elongation of aspen at the end of the active growing period. Moreover, twilight far-red light (FRL) treatment advanced the leaf bud burst of silver birch [5,6]. These examples indicate that the ratios and intensities of the individual spectral components should be controlled adequately in the plant growth chambers and glasshouses in order to obtain correct experimental results.

To perceive light signals from the sun, plants have different types of photoreceptors which include phytochromes (Phy), cryptochromes (Cry), phototropins (PHOT), and UV RESISTANCE LOCUS 8 (UVR8) [7,8,9]. Among photoreceptors, Phys (mostly phytochrome A (PhyA) and phytochrome B (PhyB)) perceive FRL and red light (RL). Phys oscillate between their active and in-active forms depending upon the R:FR ratio, which results in shade avoidance syndrome (SAS) responses [10]. Crys perceive both BL and green light (GL) signals [10,11,12]. PHOTs are also involved in the sensing of BL [13,14]. The UVR8 photoreceptor perceives UV-B radiation [15,16]. Physiological and molecular studies have shown the coordinated action of different photoreceptors, a phenomenon known as photoreceptor coaction [17]. For instance, the study on phytochrome-deficient *Arabidopsis* (*Arabidopsis thaliana* (L.) Heynh.) mutants showed that phytochrome (either A or B) is necessary for Cry1-regulated suppression of hypocotyl elongation. Further analysis revealed that Cry1-mediated BL response was inhibited in phytochrome-deficient mutants, even though Cry was unaffected in the genetic background of these mutants, as shown by western-blot analysis [18]. An earlier study on *Arabidopsis* mutants indicated that both phyA and phyB control the reaction of plants to RL, whereas BL-mediated response is also influenced by phyA and Cry1 [19]. However, further studies are required to investigate the spectral control of the co-action of photoreceptors and the involvement of the redox system in this process.

Phys and Crys control various biochemical processes through their effects on different regulatory proteins. Thus, they inactivate the photomorphogenesis repressors CONSTITUTIVELY PHOTOMORPHOGENIC1 (COP1) and phytochrome interacting factors (PIFs), which leads to the nuclear stabilization of the bZIP transcription factor ELONGATED HYPOCOTYL5 (HY5) [8]. HY5 is the master regulator of photo-morphogenesis; furthermore, it can bind to the promoter region of more than 9000 genes and directly regulates approximately 1100 of them [20,21]. HY5 can transmit light signals from a wide range of the light spectrum (e.g., UV, blue, red, FR) to light-regulated genes, including those associated with various metabolic pathways. AtHY5 has a DNA-binding domain but lacks any transcription activator domains; thus, it must cooperate with other transcription factors having such domains for the regulation of target genes [22,23,24,25]. HY5 maintains the appropriate PSII activity by controlling the balance between the oxidants and antioxidants [26,27,28], which protects the photosynthetic apparatus against oxidative stress under unfavourable environmental conditions [29,30,31]. HY5 mediates FRL induction of TANDEM ZINC-FINGER/PLUS3 (TZP) expression by directly binding to a G-box motif in the TZP promoter. Furthermore, TZP physically interacts with COP1, which inhibits COP1 interaction with HY5, ensuring its feedback regulation [32].

Almost all plant species can convert light energy into chemical one by reducing CO_2_ during photosynthesis, which ensures energy and reduces power for the whole metabolism [33]. The spectral range of sunshine, which is used for photosynthesis, is from 400 to 700 nm (known as PAR). However, plants respond to wavelengths ranging from the UV (280−400 nm) to the FR (700−800 nm) region of the spectrum, which is covered by the sensitivity of various photoreceptors. Generally, the photosynthetic rate and sugar accumulation increase linearly with photosynthetic photon flux density (PPFD), but at higher PPFDs, the formation of reactive oxygen species (ROS) is more abundant because of photo-oxidative stress, and consequently, the redox balance of plant tissues may be disturbed [33]. The most important ROS are hydrogen peroxide (H_2_O_2_), superoxide radical (O_2_^•−^), hydroxyl radical (HO^•^), and singlet oxygen (^1^O_2_). ROS may be produced in almost every subcellular compartment, except for the chloroplast-specific ^1^O_2_, and they are important signalling molecules, but their excess induces oxidative stress [34,35,36,37,38,39,40]. The antioxidant system maintains an optimal level of ROS [22] and protects cells and cell compartments against oxidative damage under various environmental stresses [41,42]. Besides the ascorbate-glutathione cycle, α-tocopherol, carotenoids, flavonoids, thioredoxins (TRXs), peroxiredoxins (PRXs), superoxide dismutase (SOD), catalase (CAT), peroxidase (POD), and glutathione S-transferases (GSTs) are the most important antioxidants [43,44,45,46,47,48,49,50]. Both ROS and antioxidants are involved in the light-dependent redox regulation of metabolism, which plays an important role in the adaptation of plants to changes in environmental conditions.

## 2. The Effect of Light Intensity and Spectrum on ROS and Antioxidants in Plant Tissues

### 2.1. Light Intensity-Dependent Changes in ROS and Antioxidant Levels

**Figure 1:**
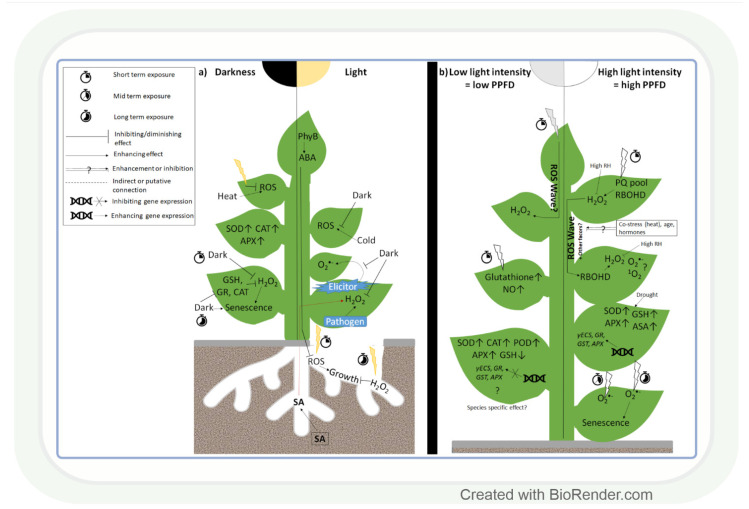
Effect of light intensity on reactive oxygen species and antioxidants. (**a**) The presence or absence of light in shoots and roots, respectively, greatly affects reactive oxygen species (ROS) accumulation and antioxidant levels under various environmental conditions. (**b**) Rapid alterations in light intensity (3–6 h) trigger a systemic ROS response (ROS wave) mediated by H_2_O_2_ and respiratory burst oxidase homolog protein D (RBOHD). Long-term response (1–7 days) of ROS homeostasis to low light or high light is also light intensity-dependent, but it is also influenced by the other environmental conditions (temperature, water availability). SOD: superoxide dismutase; CAT: catalase; GR: glutathione reductase; GSH: reduced glutathione; APX: ascorbate peroxidase; PhyB: phytochrome B; PPFD: photosynthetic photon flux density; SA: salicylic acid; ABA: abscisic acid; PQ pool: plastoquinone pool; RH: relative humidity; POD: guaiacol peroxidase; γECS: γ-glutamylcysteine synthase; GST: glutathione-S-transferase; AsA: ascorbic acid.

Changes in light intensity affect the redox environment through their effect on ROS formation and antioxidant activity (Figure 1a) [22]. While under natural conditions, the light intensity gradually increases and decreases during sunrise and sunset, respectively, in most experiments, researchers apply sudden strong changes in this parameter. Thus, the physiological and biochemical changes observed in the latter case should be interpreted carefully with respect to their role under field conditions. Interestingly, even the lack of light (2 days) can lead to ROS accumulation, as was observed in leaves of *Arabidopsis* [51]. This change was the result of a decrease in the amount of glutathione (GSH) and in the activity of glutathione reductase (GR), dehydroascorbate reductase (DHAR), and CAT. However, prolonged darkness for 4 days increased the activity of SOD, CAT, and APX enzymes in the leaves of *Pelargonium zonale* L. [52]. These results indicate that the length of the dark period strongly affects dark-induced changes in ROS and antioxidant levels. While, in leaves, the lack of light alters the redox environment in the tissues, in roots, even the very low light intensity reaching them through fractured soil layers may result in enhanced ROS accumulation (Figure 1a). This promotes root growth in the short term as a light-escape mechanism. Upon prolonged illumination, however, ROS accumulation may cause oxidative damage and reduce root length. Light-grown roots accumulated more H_2_O_2_, but not O_2_^•−^, a process that was regulated by PhyB-induced ABA signals of the shoot in *Arabidopsis*, indicating a coordinated control of ROS content in the whole plant [53]. This observation indicates that the exposure of individual plant organs to altered light intensity may also influence the ROS and antioxidant levels in other organs; therefore, the redox environment should be monitored in all organs.

Exposure to light in the case of roots and darkness in the case of shoots affects the redox environment in plants. Besides that, it is likewise strongly affected by changes in light intensity. A few hours (3–6) of exposure to high light intensity (HL) increased O_2_^•−^ accumulation and stress symptoms in *Arabidopsis* [54] and pea [55] leaves, while prolonged HL stress had a similar effect in wheat (Figure 1b) [56]. The changes in ROS levels occur immediately after the exposure to HL and provide a signalling function in order to initiate various adaptive mechanisms, as demonstrated in *Arabidopsis* [57]. Partial very short (30 min) HL illumination of *Arabidopsis* leaves exposed to low light intensity (LL) can trigger H_2_O_2_ accumulation and systemic acquired acclimation (SAA) in non-illuminated leaves as well (Figure 1b) [57,58,59]. H_2_O_2_ proved to be a systemic signal in tomato leaves, too [60]. The detected fast systemic response was termed an ‘ROS wave’. ROS waves were observed during several types of abiotic and biotic stresses, and this signal may thus have an important role in the stress adaptation process. Although H_2_O_2_ has a major role in signalling [57,61,62], ^1^O_2−_ and O_2_^•−^- responsive transcripts were also upregulated in systemic tissues of *Arabidopsis* after HL treatment [61,63]. This finding indicates that the various ROS participate simultaneously in the control of HL-inducible genes. In addition, RBHOD (O_2_^•−^ production) and the redox state of the plastoquinone pool in chloroplasts were reported to mediate SAA during HL stress [57]. ROS signals may control the response to HL together with other regulatory systems [59]. According to this assumption, ROS waves depend on phyB [64] and ABA [65], and they are mediated by vascular bundles in *Arabidopsis* [66]. Interestingly, high air humidity (85%) and heat stress mitigate systemic and local H_2_O_2_ accumulation under HL [65,66], which indicates their activating effects on the antioxidant system. However, the activation of antioxidants by light alone was also reported in several plant species, including the model plant *Arabidopsis* and the crop species wheat (Figure 1b) [55,67]. This induction was due to the transcriptional regulation of GSH metabolism-related enzymes at HL in both species [62,67]. LL increased, in turn, the activities of SOD, POD, APX, and CAT in soybean leaves (*Glycine max* L.) [68]. Based on these works, we may conclude that different ROS, in an interaction with other regulatory compounds, participate in the light intensity-dependent adjustment of the antioxidant levels. The specific role of the various ROS and antioxidants in this light (and stress) adaptation process should be clarified in further studies.

The light intensity-dependent adjustment of ROS and antioxidant levels is important in the reduction in stress-induced damages. Light promoted H_2_O_2_ accumulation and disease resistance in tobacco leaves during *Pseudomonas* infection, compared to dark conditions [69] and elicitor-induced systemic O_2_^•−^ accumulation, occurred also only in light (Figure 1a) [70]. These observations indicate that the various ROS have specific roles during the light-dependent protective processes against different biotic stresses. Although, under biotic stresses, the light-induced accumulation of ROS can be useful for killing the pathogens, the reduction in their amount would be more favourable during abiotic stresses to avoid tissue damage, as observed in *Arabidopsis* plants under heat stress [71]. The excess ROS can be removed by the efficient function of antioxidants. Pre-treatment of wheat seedlings by HL before drought resulted in an increased GSH content, which was due to the induction of *γ-glutamyl-cysteine synthase (γECS)* and *GSH synthase 2* gene expressions [61,72]. Such an effect was also shown for genes encoding various antioxidant enzymes (APX, GR, and GST). Similarly, there was a positive correlation between the activation of APX, SOD, GSH, and ascorbic acid (AsA) synthesis by HL and drought response in cashew (*Anacardium occidentale* L.) [73]. However, under extreme abiotic stress conditions, the antioxidant level can be higher due to the impairment of the antioxidant system, as shown in Cd-treated *Arabidopsis* cell culture [74] and cold-treated *Arabidopsis* leaves [75]. These results demonstrate that under controlled conditions (cultivation of horticultural plants in glasshouses), the proper adjustment of light intensity may improve stress tolerance.

### 2.2. Light Quality-Dependent Alterations in ROS and Antioxidant Levels

**Figure 2:**
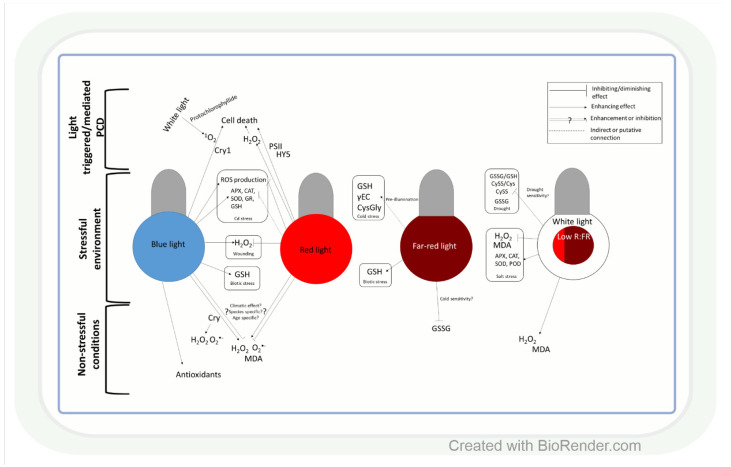
Influence of light quality on reactive oxygen species and antioxidants. Both blue and red light may act as an enhancer or a repressor of ROS accumulation, which is highly dependent on the origin (temperate or tropical climate) of the plant species, but other factors may also influence their effect on ROS. Generally, under stress conditions, blue light enhances ROS production, while red light diminishes it. ROS might have different roles in the cell death process caused by monochromic blue- or red-light illumination. The low red:far-red ratio (R:FR) exerts a beneficial effect on ROS homeostasis under stress. CRY: cryptochrome; MDA: malondialdehyde; PSII: photosystem II; HY5 elongated hypocotyl 5; SOD: superoxide dismutase; CAT: catalase; GR: glutathione reductase; GSH: reduced glutathione; APX: ascorbate peroxidase; POD: guaiacol peroxidase; γEC: γ-glutamyl-cysteine; GSSG: glutathione disulphide; CySS: cystine; CysGly: cysteinylglycine; GSSG/GSH: glutathione disulphide/glutathione ratio; CySS/Cys: cystine/cysteine ratio; PCD: programmed cell death.

Similar to light intensity, light quality may also affect ROS production through the specific effect of BL, RL, and FRL on ROS and antioxidants, as shown in several plant species (Figure 2). In leaves of *Camptotheca acuminata* seedlings, BL and yellow light, for instance, increased H_2_O_2_, O_2_^•−^ and malondialdehyde (MDA) accumulation compared to white light, whereas RL decreased their amount [76], similarly to ramie (*Boehmeria nivea* L.) leaves [77]. Conversely, in Chinese cabbage (*Brassica campestris* L. ssp. *Pekinensis Rupr*. cv. Baechoossak), BL exposure lowered H_2_O_2_ accumulation drastically compared to white light (WL) [78]. In addition, RL enhanced H_2_O_2_ production and cell death in *Arabidopsis*, and the effect was dependent on HY5-mediated light signalling [79]. These results indicate that the spectral components affect various ROS species in individual plant species differently. Therefore, general models of the regulatory mechanisms should be created very carefully. The light-responsive HY5 transcription factor (see introduction) may have a major role in this process through its many target genes. BL is more efficient than RL in the induction of its accumulation [80]. HY5 can mediate the effect of RL and BL on ROS (H_2_O_2_ or O_2_^•−^) through Phys and Crys (Figure 2) [81,82]. Furthermore, Crys indirectly participate in several ROS signalling pathways, and there are overlaps between ROS- and Cry-regulated genes [83]. During ^1^O_2_ mediated cell death in the *Arabidopsis flu* mutant (which accumulates protochlorophyllide under dark, then produces ^1^O_2_ under dark to light transition), Cry signalling and BL exposure were needed to trigger programmed cell death (PCD) after 1 h WL illumination [83].

Light quality-dependent changes in the amount of ROS also modify antioxidant levels (Figure 2). Accordingly, under low red:blue light ratio (1:3) lettuce (*Lactuca sativa* L.), plants accumulated AsA [84]. In addition, in buckwheat (*Fagopyrum esculentum* L.), BL increased the amount of total phenolics and flavonoids and the activity of antioxidant enzymes compared to RL [85]. Similar differences were found between BL and RL regarding their effects on the activities of SOD and POD and the amount of proline in ramie [77]. These observations indicate that BL seems to be more effective than RL in the induction of the antioxidant system, but this trend should be confirmed in further plant species.

The ratio of blue, red, and FR regions is very important in the adjustment of ROS and antioxidant levels to changes in the environmental conditions (Figure 2). For example, a low R:FR ratio increased H_2_O_2_ production and MDA accumulation in tomatoes under control conditions, while under salt stress, it acted oppositely on ROS formation [86]. The regulation of ROS levels by the alteration in the R:FR ratio was also confirmed during low-temperature stress in *Arabidopsis* [87], wheat, and barley [88]. Light spectrum-dependent alterations in ROS content under changing environmental conditions result in an appropriate adjustment of the antioxidant levels, which improves stress tolerance (Figure 2). In this respect, the low R:FR ratio reduced the salinity-caused damage by the induction of SOD, POD, CAT, and APX at gene expression and enzyme activity levels in tomatoes [89,90]. The spectral control of the antioxidant-dependent adaptive responses was also demonstrated in wheat [91]. Many-times lower cystine (CySS) and glutathione disulphide (GSSG) contents, as well as CySS/Cys and GSSG/GSH ratios, were detected during drought in the seedlings of the cold-tolerant wheat genotypes grown in FRL compared to those cultivated in WL, BL, or pink light. These parameters indicate that the redox environment was shifted into more reducing directions, which can ensure the appropriate function of many proteins. The involvement of FRL in the activation of AsA-GSH cycle enzymes during the cold acclimation process was shown in wheat, too [92]. Interestingly, RL increased sensitivity to Cd stress, while BL triggered the opposite effect through increasing the expression and activity of APX, CAT, SOD, and GR, as well as GSH content in cucumbers (*Cucumis sativus* L.) [93]. These results were obtained in various organs and tissues (mainly in leaves), but subcellular changes in ROS and antioxidants, which will be discussed in the next section, may be even more important in the light-dependent and redox-mediated adjustment of metabolism to the actual environmental conditions during various developmental stages of plants.

## 3. Subcellular Influence of Light Intensity and Spectrum on the Redox System

**Figure 3:**
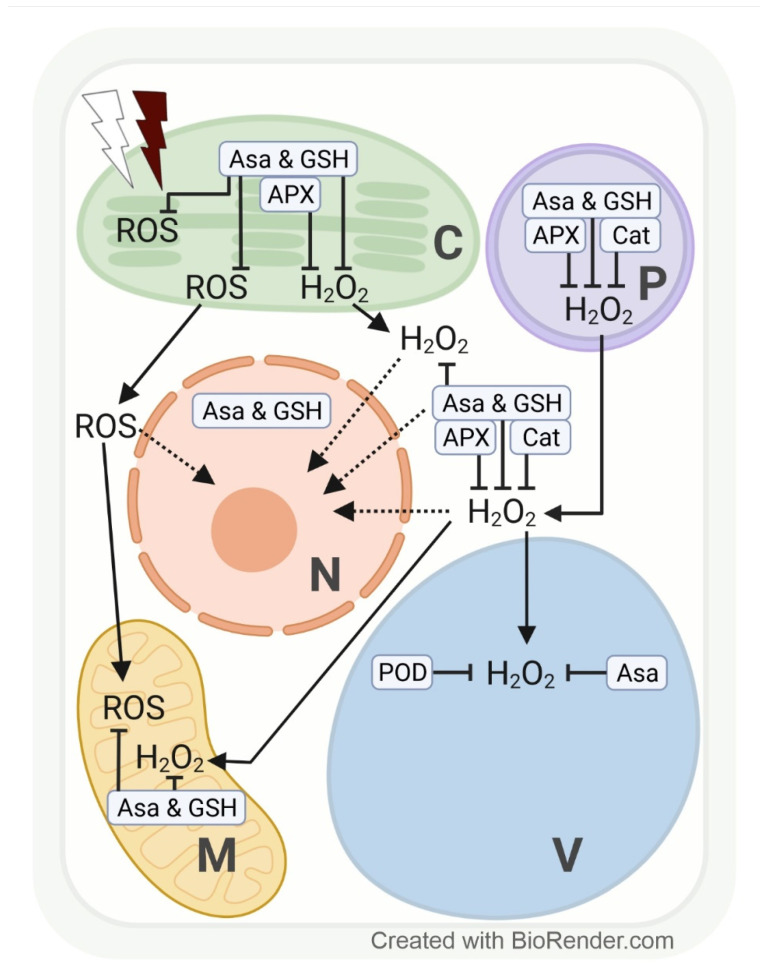
Effects of excess white and far-red light on the subcellular production of reactive oxygen species (ROS), with special emphasis on hydrogen peroxide (H_2_O_2_) and its compartment-specific detoxification by antioxidants (ascorbate, AsA; and glutathione, GSH) and enzymes in plants. Line drawing proposing a model of the effects of excess white light (white thunderbolt) and far-red light (dark red thunderbolt) on the subcellular accumulation of H_2_O_2_ (and other ROS). Possible signalling pathways are indicated by dotted arrows. APX, ascorbate peroxidase; C, chloroplast; Cat, catalase; M, mitochondria; N, nucleus; P, peroxisome; POD, guaiacol-type peroxidase; V, vacuole.

The redox system in plant cell compartments is differentially influenced by changes in light intensity and spectrum. Excess ROS accumulate particularly in the vicinity of electron transport chains, and, thus, subcellular redox adjustments are cell compartment-specific [94,95]. At the sub-organellar level, excess ROS are mainly formed close to thylakoid membranes during photosynthesis (electron transport between photosystems II and I) in chloroplasts (O_2_^•−^ and H_2_O_2_ in the stroma, ^1^O_2_ in the lumen of thylakoids) [96] or close to the inner mitochondrial membranes in the course of respiration (electron transport along the respiratory chain, Figure 3) [97].

### 3.1. Light-Intensity-Dependent Subcellular Changes in ROS and Antioxidants

The redox system in plant cell compartments reacts fast to light fluctuations, and changes in the level of various ROS may have a special regulatory function. For instance, 1 h of excess PPFD (1000 µmol m^−2^ s^−1^) increased H_2_O_2_ in genetically modified tobacco HyPer2 (fluorescent H_2_O_2_ biosensor) chloroplast stroma, cytosol, and nuclei [98]. The chloroplast-derived H_2_O_2_ accumulation in nuclei may indicate a signalling function for fast transcriptional response to HL intensity [98]. Especially under sudden light intensity changes (exposure of understory plants/leaves to full sunlight), the efficiency of electron transport between the photosystems may not be adjusted sufficiently. We may assume that the subcellular accumulation and/or distribution of H_2_O_2_ and other ROS differ in plants or leaves adapted to an environment with sudden light intensity changes according to specific coping mechanisms. However, at the cell compartment level, data addressing changes in H_2_O_2_ as a consequence of high PPFD are, to date, mainly available from model plants such as tobacco [98] and *Arabidopsis* [99]. Depending on PPFD intensity and length of exposure, Heyneke et al. [99] showed that in *Arabidopsis*, excess H_2_O_2_ from overstrained electron transport chains in chloroplasts and from photorespiration in peroxisomes subsequently accumulates in the cytosol and later in the vacuole. Similarly, a transient increase in H_2_O_2_ (detected via roGFP2-Orp1) and glutathione redox potential (*E*_GSH_) (detected via Grx1-roGFP2) upon photo-oxidative stress was detected in chloroplasts, followed by the cytosol and mitochondria in genetically modified *Arabidopsis* [100,101]. We may expect a subsequent increase in H_2_O_2_ in vacuoles, which, however, requires the development of a vacuole-targeted biosensor line. Further, ^1^O_2_ accumulates in chloroplasts when electron transport is inefficient under high PPFD stress, which leads to photo-inhibition by irreversible damage to photosystem II.

The subcellular redox system is particularly strained under a combination of stresses, such as high PPFD and osmotic stress. Closure of the stomata (to prevent water loss) limits the availability of the electron acceptors ADP and NADP and the regeneration of 3-phosphoglycerate in chloroplasts. To prevent electron transfer overload and ROS formation close to thylakoid membranes, ADP and NADP (and 3-phosphoglycerate for primary metabolism) are provided by photorespiration. A side reaction of photorespiration, however, increases the H_2_O_2_ concentration in peroxisomes. Limited ADP availability likewise contributes to an above-normal ROS (initially O_2_^•−^, followed by H_2_O_2_) accumulation in mitochondria (Figure 3) [101,102,103,104].

Under high PPFD stress, H_2_O_2_ accumulation in chloroplasts can be scavenged by AsA, GSH, and APX activity, in the cytosol and peroxisomes by AsA, GSH, APX, and CAT activities, and in vacuoles by AsA and POD (EC 1.11.1.7) activities (Figure 3) [99,105,106,107]. AsA concentration rises with increasing light intensity, and AsA recycling has been localized in peroxisomes, mitochondria, and the cytosol [108]. Overexpression or silencing of MDHR3 (monodehydroascorbate reductase 3, one of the iso-enzymes which catalyse the regeneration of AsA via oxidation of GSH) in tomatoes resulted in lower or higher AsA concentrations, respectively. Fluorescent targeting with GFP and mCherry suggests a dual localisation of MDHR3 in peroxisomes and the cytosol [109].

Darkness and light strongly influence the subcellular glutathione level, as shown by its diurnal rhythm in *Arabidopsis* mesophyll cells, reaching a maximum after 3 h of light period and a minimum at the end of the night [110]. This much lower glutathione concentration seems to be resulting from limited availability of the glutathione precursors glycine (from reduced photorespiration) and cysteine (from reduced sulphur uptake and incorporation) during darkness [111,112,113]. Interestingly, 24 h of darkness depleted *Arabidopsis* glutathione levels in mitochondria (84%) and peroxisomes (53%), whereas AsA labelling density remained comparatively stable [51]. Results from low PPFD intensity experiments corroborate these observations.

Similar to darkness, low PPFD also strongly reduced the glutathione levels in peroxisomes of *Arabidopsis* and in peroxisomes, nuclei, and the cytosol of wheat [99,107]. In wheat, the glutathione concentration under low PPFD may have remained unchanged in chloroplasts at least partly due to a transcriptional upregulation of the chloroplast-localized γ-ECS [107]. Subcellular AsA concentrations in *Arabidopsis* were largely unaffected by low PPFD, irrespective of the length of treatment [99], which can be explained by the larger size of the ascorbate pool.

High PPFD for a few hours stimulated total glutathione accumulation in wild-type *Arabidopsis*, particularly in compartments of glutathione synthesis (chloroplasts and the cytosol) and of ROS and H_2_O_2_ detoxification from photorespiration (peroxisomes). High PPFD in the short term, by contrast, increased the mitochondrial glutathione concentration drastically (900%) in the glutathione-deficient Arabidopsis *pad2-1* mutant, probably to ensure efficient respiration under high PPFD stress [99]. The compartment-specific increase in total glutathione in wheat was accompanied by the transcriptional upregulation of cytosolic GR [107]. GR plays an important role in the adjustment of the optimal GSSG/GSH ratio, which determines the half-cell reduction potential of this redox pair [114]. Using redox-responsive GFP, a gradual increase in *E*_GSH_ was reported in potato chloroplasts with increasing light intensity. Interestingly, chloroplasts were able to return to a normal level of glutathione oxidation state after a 14 h treatment at 1250 µmol m^−2^s^−1^, but not if high PPFD was combined with cold stress (3 °C) in mature leaves, conditions favouring PSI photoinhibition [114]. The sensitivity of chloroplasts to a combination of high PPFD and cold (or other osmotic stresses) certainly depends on the natural habitat of a plant.

### 3.2. Light-Spectrum-Dependent Subcellular Changes in ROS and Antioxidants

Besides light intensity, the light spectrum is also an important regulator of subcellular ROS and glutathione levels. ROS formation in *Arabidopsis* chloroplasts occurs under continuous FRL as a consequence of impaired chlorophyll synthesis and accelerates when seedlings are transferred from continuous FRL to WL with a normal R:FR ratio due to inefficient electron transfer between the photosystems. Adjustments to WL after transfer from continuous FRL may be facilitated by H_2_O_2_ signalling in wild-type *Arabidopsis* [115,116]. In mature leaves transferred from WL with a normal R:FR ratio to light with a decreased R:FR ratio (shade), ROS and H_2_O_2_ accumulation in the short-term results from an imbalanced excitation state of photosystems II and I. Under long-term growth in FR enriched spectrum (shade), ROS and H_2_O_2_ concentrations in chloroplasts rise as a consequence of the limited availability of reductants (NADPH and ATP) and assimilates. H_2_O_2_ may first accumulate in chloroplasts and peroxisomes and subsequently in the cytosol and the vacuole (Figure 3) [102,115,116,117,118].

Total glutathione concentration in wild-type *Arabidopsis* and the *vtc2-1* mutant was largely unaffected by a decreased R:FR ratio (blue/red 1:5; R:FR: 10:1) at 250 µmol m^−2^s^−1^ compared to WL (blue/red 1:2; R:FR: 15:1) of the same intensity. By contrast, in the glutathione-deficient *pad2-1* mutant, the total glutathione concentration increased in nuclei by 200%, in peroxisomes and the cytosol by 100%, and in wheat cytosol by 180%, by 150% in chloroplasts, and by 100% in nuclei and peroxisomes [107]. These results indicate that the subcellular redox system in *Arabidopsis* and wheat plants is controlled by the R:FR ratio. It seems that the adaptations in subcellular glutathione distribution are similar under high PPFD and a decreased R:FR ratio in *Arabidopsis pad2-1* and wheat (Figure 3) [107]. For redox adjustments to increased PPFD, a high nuclear GSH demand seems to play a role, whereas a re-location of glutathione to peroxisomes seems to facilitate adaptations to FRL under AsA deficiency [99,107]. These are only first insights into how the subcellular redox system in plants responds to changes in spectral composition, much is yet to be discovered. Based on the results obtained from leaf tissues [59,67], BL and other spectral components may also influence the subcellular glutathione distribution and, subsequently, other redox-sensitive metabolic processes in plants.

## 4. Light Intensity- and Spectrum-Associated Adjustment of Metabolism

Changes in light conditions result in shifts in primary and secondary metabolism [119,120,121]. The redox system plays an important role in the mediation of changes in light intensity and spectrum on carbon, nitrogen, sulphur, lipid, nucleic acid, and secondary metabolism. Generally, the effect of light quality on most aspects of plant metabolism has received less attention than that of light intensity.

### 4.1. Light Control of Carbohydrate Metabolism

#### 4.1.1. Light Intensity-Associated Regulation of Carbohydrate Metabolism

**Figure 4:**
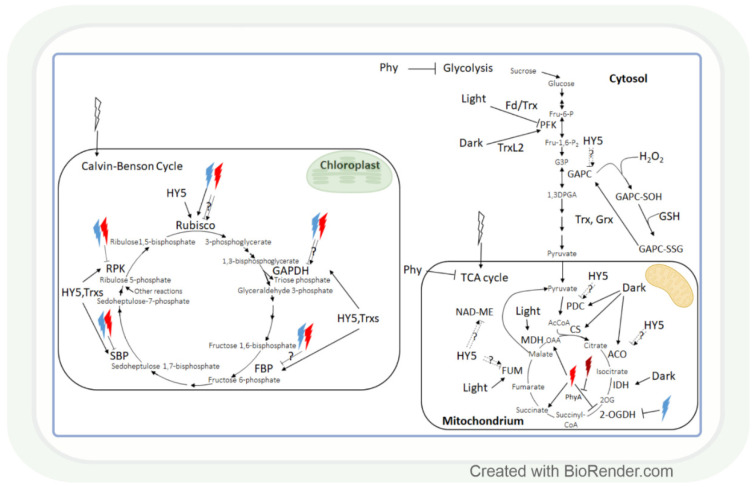
The effect of light intensity and spectrum on carbohydrate metabolism. Monochromic red or blue lights may inhibit or enhance the enzymes of the Calvin–Benson cycle. Most of these enzymes are HY-5 and/or Trx-regulated. Interestingly, several enzymes of carbohydrate catabolism are light repressed, while others are light enhanced. Light-associated redox regulation of the Calvin–Benson cycle is better known than in the case of tricarboxylic acid (TCA) cycle. Rubisco: ribulose-1,5-bisphosphate carboxylase oxygenase; GAPDH: glyceraldehyde-3-phosphate dehydrogenase; FBP: fructose-1,6-bisphosphatase; Fd: ferredoxin; SBP: sedoheptulose-1,7-bisphosphatase; RPK: ribulose-5-phosphate kinase; Trx: thioredoxin; HY5: elongated hypocotyl 5; HL: high light; GAPC: cytosolic GAPDH; GAPC-SOH: oxidised GAPC; GAPC-SSG: glutathionylated GAPC; Grx: glutaredoxine; PFK: phosphofructokinase; TrxL2: Trx-like2; MDH: malate dehydrogenase; NAD-ME: NAD-dependent malic enzyme; FUM: fumarase; CS: citrate synthase; IDH: isocitrate dehydrogenase; ACO: aconitase; PDC: pyruvate dehydrogenase complex; 2-OGDH: 2-oxoglutarate dehydrogenase; OAA: oxaloacetate; 2OG: 2-oxoglutarate; PhyA: phytochrome A; AcCoA: acetyl-CoA; Fru-6-P: fructose-6-phosphate; Fru-1,6-P_2_: fructose-1,6-bisphosphate; G3P: glyceraldehyde-3-phosphate; 1,3DPGA: 1,3-diphosphoglycerate.

Plants have the ability to produce carbohydrates by using light energy and water to convert CO_2_ to different types of carbohydrates via photosynthesis [122]. NADPH ensures the reducing power for this process; therefore, it depends on the cellular redox environment (Table A1). There are 5 important enzymes in the Calvin–Benson cycle (CBC), which are strongly regulated by light, namely: ribulose-1,5-bisphosphate carboxylase oxygenase (Rubisco), glyceraldehyde-3-phosphate dehydrogenase (GAPDH), fructose-1,6-bisphosphatase (FBP), sedoheptulose-1,7-bisphosphatase (SBP), and ribulose-5-phosphate kinase (RPK) (Figure 4). These enzymes are also controlled by the transcription factor HY5, coordinating carbon assimilation with other metabolic processes at the transcriptional level [123]. In the shoot, HY5 promotes carbon assimilation and translocation, whereas, in the root, it activates NRT2.1 expression and nitrate uptake. It has been shown in *Arabidopsis* mutants that HY5 is a shoot–root mobile signal that coordinates shoot growth and carbon assimilation with root growth and nitrogen uptake in different light environments [124,125,126,127].

HY5 is a master regulator of many genes, including those that affect the redox state in tissues. Thus, it can mediate the light regulation of CBC through the ferredoxin/thioredoxin (Fd/Trx) system to which the enzymes of CBC are linked, except for Rubisco (Figure 4). Ferredoxin (Fd) determines the distribution of electrons to distinct processes, including the production of NADPH and redox signalling for the activation of CBC enzymes via the ferredoxin-dependent thioredoxin reducing system. The Fd/Trx system transfers reducing equivalents to various proteins containing redox-active cysteine residues, thereby modulating their activity [128,129,130]. As it has been already discussed in Section 2.1, the HL illumination of a distant leaf could launch an ROS wave across the plant, which prepares CO_2_ assimilation in a non-illuminated leaf for the upcoming illumination. During this process, H_2_O_2_ as an oxidant may control the activity of CBC through the oxidation of thioredoxins. The HL sensitivity of the enzyme activities of CBC was shown in the *Arabidopsis* chloroplast glucose-6-phosphate/phosphate translocator mutant, *gpt2*, which does not acclimate to HL [131]. In this mutant HL activation of RPK, Rubisco small subunits (Rbcs 1A and 3B), phosphoglycerate kinase 1 (PGK1), GAPDH B, triose phosphate isomerase (TPI), transketolase 1 (TKL1), SBP, ribulose 5-phosphate epimerase (RPE), and ribose 5-phosphate isomerase (RPI) were diminished, too. Interestingly, the short-term exposure (15-20 min) of the dicot *Arabidopsis* and monocot rice to increasing light intensities affected the CBC differently in the 2 species [132]. The balance between subprocesses was different since 3-phosphoglycerate reduction was favoured in *Arabidopsis*, and ribulose 1,5-bisphosphate regeneration was favoured in rice in HL. These observations indicate that the results obtained in *Arabidopsis* cannot be generalized, and several plant species should be used for the creation of models describing the metabolic effect of light intensity.

Similar to carbon assimilation, carbohydrate catabolism also changes with alterations in the light conditions. The effect of light-induced redox changes on glycolysis was shown in the case of GAPC, a key enzyme in glycolysis, since it was inactivated by H_2_O_2_-mediated oxidation [133]. GAPC-SOH may undergo glutathionylation by GSH, which would lead to the formation of GAPC-SSG. GAPC-SSG may be restored to GAPC by Trxs or glutaredoxins (Grxs) [133]. Another glycolytic enzyme, phosphofructokinase (PFK), is deactivated via the Fd/Trx pathway during the light-to-dark transition in *Arabidopsis* and activated by the Trx-like 2 (TrxL2) pathway during the night [134]. Since Trxs regulates not only the enzymes of CBC but also the enzymes of the TCA cycle, glycolysis, and photorespiration [35,135], these pathways may be involved in the coordination of these processes.

Generally, most studied metabolites of the tricarboxylic (TCA) cycle were increased under HL, and this effect was redox-dependent [136]. Correspondingly, modifying the redox environment by antisense expression of pumpkin or tobacco ascorbate oxidase expression decreased malate accumulation and alleviated the HL enhancing effect on fumarate in tobacco plants. The effect of HL on the TCA cycle is mediated by the HY5 transcription factor, as shown for the pyruvate dehydrogenase complex (PDC), aconitase, fumarase (FUM), and NAD-dependent malic enzyme (NAD-ME) (Figure 4) [123]. During the TCA cycle, the enzyme activities of PDC, citrate synthase (CS), aconitase, and NAD-dependent isocitrate dehydrogenase (IDH) were dark-enhanced, and FUM and malate dehydrogenase 2 (MDH2) activity increased during the day. Interestingly, NAD-ME activity changed during the light-to-dark and dark-to-light transitions [137]. The light control of the activity of these NAD-dependent enzymes can be likewise mediated by the mitochondrial alternative oxidase (one of the terminal oxidases of the plant mitochondrial electron transport chain which are also light-responsive) [138].

#### 4.1.2. The Effect of Light Quality on Carbohydrate Metabolism

The light spectrum is also an important regulator of carbohydrate metabolism since the low R:FR ratio increased this process by the induction of photosynthetic capacity in soybean seedlings [139]. The observed change may be a result of the activation of Rubisco, the key enzyme of carbon assimilation (Figure 4). This hypothesis was confirmed for other spectral ranges in tomato and sweet pepper [27,140,141] since BL- and red-blue-light (RBL)-grown plants had increased amount/activity of Rubisco compared to WL grown plants. However, the use of RL alone resulted in a lower amount or decreased activity of Rubisco [27,140,141], indicating the specific effect of different spectral ranges and their ratios on Rubisco. GAPDH activity was also enhanced by BL and RBL, and it was decreased by RL in sweet pepper and tomato [27]. However, Li et al. [140] found in tomato seedlings that GAPDH activity was significantly increased by monochromatic RL treatment and decreased in monochromatic BL and also in RBL compared to WL. These conflicting results may be explained by the species–specific effect of the various spectral ranges, and the developmental stage and cultivation conditions may also modify their influence. This presumption is also confirmed by the effect of RL and BL and their mixture on the activities of FBP, SBP, and RPK in various plant species [140,142,143].

Carbohydrate catabolism was also influenced by the light spectrum since the compounds of the TCA cycle were enriched among the 99 metabolites (DEMs) (42 upregulated and 57 downregulated) detected in different amounts in RL and BL in *Mesona chinensis* [144]. Some RL-responsive metabolites from the TCA cycle like succinate or oxaloacetate, participating in the stomatal opening, were also identified in *Arabidopsis* [145]. In addition, the activity of the key TCA cycle enzyme, 2-oxoglutarate dehydrogenase (2-OGDH), was also influenced by the light spectrum (Figure 4) [146]. Both BL and RL could diminish 2-OGDH activity in maize, but RL was more efficient, and its effect could be reversed by FRL. A different transcriptional regulation of 2-OGDH isoenzymes was also shown in response to RL and BL in maize and *Arabidopsis*. In *Arabidopsis phyA* knockout mutants, the effect of RL was reduced only on *OGDH2* transcription, but *phyB* mutants showed a wild type-like expression of *OGDH1* and *OGDH2*, which observation indicates the specific regulatory effects of various phytochromes on this enzyme [146].

### 4.2. Light Regulation of Nitrogen Assimilation and Amino Acid Levels

**Figure 5:**
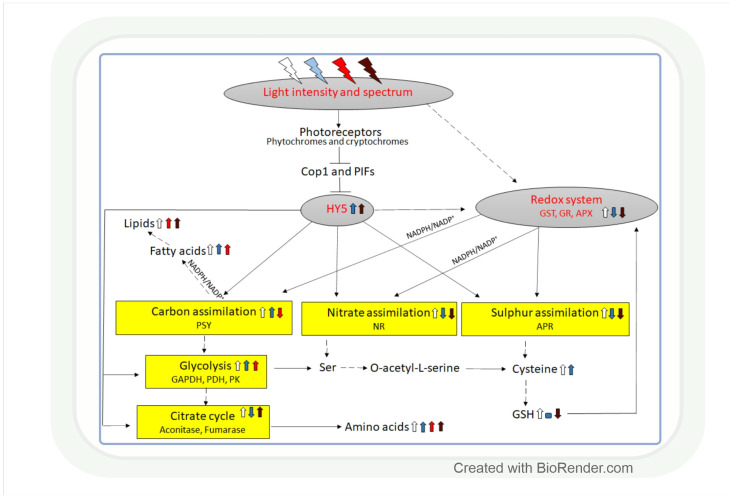
Light intensity- and spectrum-associated coordination of carbon, nitrate, and sulphate assimilation. The control of the three metabolic pathways is interconnected through the effect of light on the redox system and the HY5 transcription factor. The redox system regulates them through the NAD(P)H/NAD(P)^+^ and GSH/GSSG redox couples and HY5 through its binding to the promoter of the genes related to these processes. White, blue, red, and dark red arrows indicate the effect of white, blue, red, and far-red light, respectively. Upwards arrows: induction, downwards arrows: repression, horizontal line: no effect. Continuous lines: direct connection, dashed lines: indirect connections with additional intermediates. APR: adenosine phosphosulphate reductase, APX: ascorbate peroxidase, Cop1: constitutive photomorphogenic1, GAPDH: glyceraldehyde phosphate dehydrogenase, GR: glutathione reductase, GST: glutathione S-transferase, NR: nitrate reductase, PDH: pyruvate dehydrogenase, PK: pyruvate kinase, PIF: phytochrome-interacting factor, PSY: phytoene synthase.

Light intensity and spectrum control carbon, nitrate, and sulphate assimilation in a coordinated way through the involvement of the redox system and HY5 (Table A1 and Figure 5). Nitrate reduction depends on the availability of NAD(P)H; therefore, it is influenced by light-dependent redox processes. The reduced form of nitrogen will be incorporated into amino acids. Light intensity and spectrum may affect the amount of free amino acids through their influence on carbon fixation and nitrate reduction, as discussed in the previous section for the former one. While, in these processes, carbon and nitrogen are incorporated into organic compounds, intermediates of glycolysis and the TCA cycle serve as starting points for the synthesis of members of the various amino acid families.

#### 4.2.1. Light Intensity-Associated Changes in Nitrogen Assimilation and Amino Acid Levels

A coordinated upregulation of carbon assimilation and nitrate reduction was induced by high PPFD in cotton, which supports the regulatory model shown in Figure 5 [147]. The effect of light on nitrate reduction was also confirmed in maize, in which nitrate reductase activity exhibited a diurnal rhythm with 2–3-fold higher activity during the light period compared to the dark (Figure 5) [148]. The light-dependent transcriptional regulation of this enzyme was observed in *Arabidopsis* [149]. In *Arabidopsis* loss-of-function mutants, it was shown that the HY5 and the ELONGATED HYPOCOTYL 5-HOMOLOG (HYH) transcription factors are involved in this process [7,97,150,151]. In addition, the light signalling-related COP1 and PIF transcription factors, promoting the degradation of HY5 and HYH, also participate in the regulation of nitrate reductase activity in *Arabidopsis* (Figure 5) [150]. HY5 may have a similar function in the light-dependent control of nitrate reduction in the monocot crop species, such as maize, wheat, and rice, but this hypothesis should be corroborated in future studies. Besides HY5, the NAD(P)H/NAD(P)^+^ redox couple is also involved in the adjustment of nitrate reduction to the changes in the light intensity through its effect on the redox state of the plant tissues [152].

The activation of nitrate reduction by HL allows an increased incorporation of nitrogen into Gln and other amino acids. Accordingly, HL resulted in a coordinated strong increase in several free amino acids (Asp, Pro, Val, Ala, Thr, Tyr, Ile, Phe, Hys, Cys) in *Chlamydomonas reinhardtii*, as shown by the high positive correlations between the changes in their levels (Figure 5) [153]. Simultaneous increase in free amino acid and GSH levels was observed during the light period compared to the dark in transgenic poplar overexpressing γGluCys synthetase (rate-limiting in GSH synthesis) in chloroplasts which indicates the participation of the redox system in the mediation of the effect of light on amino acid synthesis [154]. A positive relationship between the amount of free amino acids and light intensity was also observed in wheat seedlings except for Pro, Met, Thr, ornithine, and cystathionine [67]. Light intensity regulates free amino acid concentration at the transcriptional level, as shown by the increased expression of the genes related to Arg, Glu, Asp, and Ser metabolism. Since Arg is the precursor of polyamines, its light-induced accumulation activates their synthesis, as demonstrated with elevated putrescine and spermidine contents at higher PPFDs in leaves of wheat [155]. Polyamines, together with certain free amino acids (Pro, Glu, Cys), may have a role in the adaptation to changes in light conditions through their associations with the redox system. The light-induced increase in free amino acid levels was not accompanied by a change in the soluble protein content, indicating that free amino acid accumulation was not a result of protein degradation in *Arabidopsis* [113].

The induction of amino acid accumulation by HL may also be important in stress response since several amino acids participate directly (Pro–osmoprotectant) or indirectly (as precursors of GSH and polyamines) in the reduction in the stress-related damages. Indeed, the light intensity had a very large effect on the drought-induced accumulation of Pro, an amino acid that is very important in the adjustment of osmotic pressure and the redox environment during drought stress in plants [156]. Cultivation of wheat at various light intensities affected the amino acid metabolism at transcript and metabolite levels during the subsequent drought period since both increased with increasing light intensities [72]. This effect was observed in the drought-tolerant genotype after a moderate increase in light intensity, whereas, in the sensitive one, it was only after a strong increase in light intensity, indicating a species-specific reaction.

#### 4.2.2. The Effect of Light Quality on Nitrogen Assimilation and Amino Acid Levels

Similar to light intensity, the light spectrum can also affect nitrate reduction through its effect on the HY5 transcription factor or the redox system (Figure 5). Comparing the effect of WL, and monochromatic BL and RL, the nitrate reductase and nitrite reductase activities and the expression of the genes of these enzymes were the highest in BL-treated pakchoi plants (*Brassica campestris* L.) [157]. Transcriptional regulation of nitrate reduction by supplementary BL and FRL was observed in wheat [67]. Spectrum-dependent redox control of the nitrate reduction was suggested in wheat based on simultaneous changes in the GSH/GSSG ratio in leaf tissues [67].

The spectrum-dependent control of nitrate reduction allows the subsequent modification of the free amino acid levels, as shown for supplementary FRL, which increased the amount of Glu, Gln, and Pro in flag leaves of wheat [158]. It should be noted that, similar to light intensity, spectral changes likewise did not affect the total protein levels in flag leaves of wheat; therefore, the observed changes in their levels were not derived from protein degradation [158]. An inducing effect of BL on free amino acid accumulation was shown in barley [159]. Specific ratios of red, blue, and green spectral components seem to be important in the control of free amino acid levels, based on the results obtained in lettuce [160]. Interestingly, the low R:FR ratio in shade induced the accumulation of several amino acids (Glu, Pro, Gly, Asp, Val, Ser, Glu, Thr) and the related transcripts in leaves of tea compared to its leaves exposed to sunshine [161]. This result differs from the observations obtained in other plant species. Given that tea plants in natural habitats are adapted to shade, it should be considered that the decreased free amino acid levels in sunshine indicate a disturbed metabolism.

Similar to light intensity, light spectrum was also involved in the adjustment of amino acid levels to adverse environmental conditions (Figure 5). Namely, BL and FRL participated in the transcriptional control of salt-induced Pro accumulation by inducing its synthesis and repressing its degradation in *Arabidopsis* [162]. This regulation is based on the interaction of HY5 with the promoter region of the genes of Pro metabolism. In addition, supplementary BL or FRL strongly increased the level of several free amino acids during drought, only in a tolerant wheat variety, which indicates the relationship between their spectrum-dependent changes and adaptation processes [91].

### 4.3. Light Regulation of Sulphur Assimilation and Glutathione Metabolism

#### 4.3.1. Light Intensity-Dependent Control of Sulphur Assimilation and Glutathione Metabolism

Sulphate reduction, having a diurnal rhythm, exhibits a great sensitivity to light, as shown by the 5–10-fold increase in the activity of its key enzyme, adenosine phosphosulphate reductase (APR), during the light period compared to the dark in leaves of 2 maize varieties at seedling stage (Figure 5) [148]. The light activates this enzyme at the transcriptional level, as shown in *Arabidopsis*, resulting in the increased incorporation of ^35^S from sulphate into cysteine and GSH [163]. A light-dependent control of sulphate reduction was also observed in wheat at both transcript and metabolite levels [67]. Interestingly, the increase in light intensity had an activating effect on these parameters. The bZIP transcription factor HY5 participates in the transcriptional regulation of APR through its direct binding to the APR1 and APR2 promoters, which was confirmed by chromatin immunoprecipitation in *Arabidopsis* (Figure 5) [164]. In addition, a similar regulation of a sulphate transporter (AtSULTR1;2), another key gene of sulphate assimilation regulated by HY5, was observed in *Arabidopsis* [164]. The PHYTOCHROME AND FLOWERING TIME 1 (PFT1) protein may be involved in this process by bridging HY5 with other transcription factors during the light-dependent control of APR [165].

The induction of sulphate reduction by HL allows the accumulation of its end product, cysteine, which is a precursor of GSH and has a central role in the redox-dependent regulation of metabolic pathways. Accordingly, an increase in cysteine content resulted in a simultaneous accumulation of GSH with increasing light intensity in wheat (Figure 5) [67]. This change derived from transcriptional activation of GSH synthesis, as indicated by an increase in *glutathione synthase* expression. Interestingly, the larger glutathione pool became more reduced at increased light intensities due to the unchanged level of GSSG and the increased transcriptional activation of the enzymes of the ascorbate glutathione cycle (GR, APX). These changes, in turn, alter the redox environment of tissues and consequently many metabolic pathways [152].

HL can induce the GSH-dependent protective mechanisms against environmental stresses, as was observed in wheat subjected to drought [72]. With increasing light intensity, the amount of GSSG and its ratio compared to GSH increased, which resulted in a better recovery of the seedlings grown under HL. These results show that moderate oxidative stress induced by HL could reduce the negative effect of drought. The GSSG/GSH ratio strongly depends on the activity of GR and APX (reducing GSSG in the ascorbate-GSH cycle), enzymes which were induced by HL at the transcriptional level in wheat [67]. The activation of APX activity by HL was observed in the leaves of apple trees, too [166]. Besides GR, GSTs also have a strong influence on the redox state of the glutathione pool since they reduce the amount of GSH by catalysing GSH conjugation to electrophilic compounds [167]. The more soluble derivatives can more easily enter the vacuoles, where they can be stored or converted to other metabolites. The expression and activity of GSTs are reduced by LL and darkness, and they are increased by HL [72,167]. The light-induced activation of GSTs may reduce the harmful effect of abiotic stresses through the detoxification of lipid peroxides by their conjugation with GSH.

#### 4.3.2. Light Quality-Associated Regulation of Sulphur Assimilation and Glutathione Metabolism

Sulphate reduction was induced by supplementary BL and was not affected by FRL, based on the amount of *APR* expression and the amount of Cys in wheat (Figure 5, Table A1) [67]. This effect is probably mediated by HY5, which depends on spectral conditions. BL proved to be more efficient than RL in the activation of HY5 at the transcriptional and post-transcriptional levels in *Arabidopsis* [80]. The ratio of the blue and red components in the spectrum regulates the binding of HY5 to the specific elements in the promoter of its target genes. HY5 may be responsible for the coordinated light-dependent transcriptional control of many metabolic pathways, including sulphate, nitrate, and carbon assimilation, which are interconnected by O-acetylserine (Figure 5) [124,165,168].

As described for sulphate reduction, glutathione metabolism was also affected by the light spectrum (Figure 5). Supplementary BL did not affect the amount of GSH, whereas supplementary FRL even decreased it compared to WL in wheat, and both spectral modifications reduced the transcription of the *glutathione synthase* gene [67]. The transcriptional regulation of the genes of GSH-metabolism by spectrum was also confirmed in maize, in which BL had a stronger effect on their expression compared to RL [169]. GSH, due to its central position in the redox system, may mediate the effect of the light spectrum on many metabolic processes, but the clarification of these regulatory mechanisms needs further investigations.

Light spectrum-associated changes in the size and the redox state of the glutathione pool may affect the response to various abiotic stresses. This hypothesis was not confirmed in wheat since the reduction in the GSSG/GSH ratio by FRL was not accompanied by changes in the recovery after drought in a drought-tolerant wheat genotype [91]. Similarly, the growth of wheat seedlings in supplementary FRL also affected the redox state of the glutathione pool during cold treatment, but it did not alter the recovery of the plants compared to their cultivation in WL [92]. The change in the redox state of the glutathione pool may not derive from the transcriptional regulation of the genes involved in GSH synthesis as observed in wheat and *Arabidopsis* [170]. However, the enzymes involved in the reduction of GSSG or conjugation of GSH to other molecules may be controlled at the gene expression level. Accordingly, the transcription of the *GR* gene was repressed by supplementary FRL in wheat [67]. The expression and activity of GSTs are increased by RL [72,167]. The involvement of a GST in phytochrome A-mediated signalling was demonstrated through its interaction with the FR insensitive 219 protein in *Arabidopsis* [171]. In addition, an HY5-controlled GST was identified in *Arabidopsis* [123]. GSTs, in turn, can modify the metabolic processes through the glutathionylation of their target proteins.

### 4.4. Light Control of Lipid Metabolism

#### 4.4.1. Light Intensity-Associated Alterations in Lipid Metabolism

In plants, de novo fatty acid synthesis takes place mostly in the chloroplasts of green tissues or in plastids of non-photosynthetic organs and limitedly in the mitochondria, and most enzymes are affected by light conditions (Table A2). Accordingly, fatty acid and membrane lipid synthesis were significantly increased in LL compared to control conditions, while the opposite relationship was observed between HL and control-condition-grown *Arabidopsis* [172]. In addition, it was shown that light intensity-dependent changes in fatty acid synthesis derived from the post-translational regulation of the plastidic acetyl-CoA carboxylase activity. The effect of light intensity on lipid synthesis is mediated by the redox system. Thus, the formation of fatty acids from 3-phosphoglycerate (a component of CBC, a link to carbon assimilation) is an NADPH-consuming process [152]. In addition, members of lipid/fatty acid biosynthesis (Malonyl CoA:ACP transacylase, MCAT; Acetyl-CoA biotin carboxyl carrier) are targets of modification by Trx, Grx, and GSH [173]. Among the lipid biosynthetic enzymes, the light and redox control of ACCase, the Trx regulation of monogalactosyl diacylglycerol synthase (MGD), and the Prx Q requirement of fatty acid desaturase 4 (FAD4) were shown in *Arabidopsis* [174]. Light control of *AtFAD7* was demonstrated in *Arabidopsis* since its promoter contains a light-responsive element [175]. Further evidence for the light regulation of FADs has been reported in cotton (*Gossypium hirsutum*) [176] and soybean cell suspension [177]. In this process, the light-responsive HY5 transcription factor participates since it targets many genes involved in lipid biosynthesis, for example, digalactosyldiacyl glycerol synthase 1 (DGD1), FAD3, or FAD6 [123]. The light-dependent transcriptional control of triacylglycerols (main components of plant oils) was shown in olive plants since the *phospholipid: diacylglycerol acyltransferase* gene was downregulated under darkness [178]. Further evidence for the light control of lipid formation was obtained by the study of its diurnal changes [179]. Thus, polar lipid fatty acid biosynthesis occurs in the light period of the diurnal cycle, whereas fatty acid desaturation takes place in the dark period in *Arabidopsis.* Furthermore, membrane galactolipid, phospholipids, and lysophospholipid profiling showed interesting diurnal regulation of the plant lipidome, highlighting the role of phospholipase D [179]. Similar to *Arabidopsis*, phospholipid levels were also affected by light in barley since the expression of genes encoding the components of the phospholipid signalling pathway follows a circadian rhythm [180]. The results obtained in dicot and monocot species indicate the general role of light in the regulation of phospholipid levels in plants.

Changes in light intensity are useful for the modification of lipid composition in order to increase stress tolerance of crops cultivated in glasshouses or on vertical farms. Both HL and LL proved to be appropriate for this purpose in *Arabidopsis* [55,181] and olive callus cultures at the control growth temperature [182]. In both species, differences were found in lipid composition between HL, ambient light, and/or LL. In *Arabidopsis*, ACCase activity increased in LL compared to normal light and HL, whereas total lipid content was also higher under LL than in normal light and HL caused a decrease in total lipid level. However, the authors detected no consistent changes in the protein abundance of ACCase subunits, which indicates a post-translational regulation of the enzyme [55]. Similar to the control growth temperature, light conditions also affected the lipid composition at high temperatures based on the differences between the amounts of several lipid species in light and darkness in *Arabidopsis* [181]. High temperature and HL stresses are often combined under natural conditions, and under such conditions, tolerance mechanisms are based on the appropriate modulation of photosynthesis and membrane integrity [183,184,185]. Thus, the modification of membrane lipid composition based on the results obtained in growth chambers may be appropriate to reduce the yield loss of crops.

#### 4.4.2. Light Quality-Associated Changes in Lipid Metabolism

Besides the intensity of light, its spectrum is also involved in the regulation of lipid metabolism (Table A2). The RL and FRL ranges, for instance, have an important role in the control of MGDG formation in cucumber and *Arabidopsis* [23]. In addition, a low R:FR ratio modified the expression of the *phosphatidylinositol 4-kinase* (*HvPI4K*) gene under light/dark cycles in barley (*Hordeum vulgare* L.) [180]. Interestingly, high BL influenced the expression of many genes involved in fatty acid biosynthesis, and the accumulation of several lipid metabolites differed under low, moderate, and high BL in tea [186]. These observations indicate that the light spectrum controls the lipid composition at the transcriptional level. The effect of light spectrum on lipid composition had been studied more intensively in microalgae than in higher plants. Sharma et al. (2020) reported that in *Phaeodactylum tricornutum*, RL increased lipid content compared to WL control [187]. The level of polyunsaturated fatty acids (PUFAs) increased, whereas that of monounsaturated fatty acids decreased. In *P. tricornutum*, Duarte et al. (2021) reported that BL resulted in a higher production of total fatty acids, namely saturated TAGs [188]. RL, in turn, increased the cells’ hexadecatrienoic acid (HTA) and eicosapentaenoic acid (EPA) content. In addition, GL treatment decreased polyunsaturated lipid content in four microalgae species [189]. Based on these results in microalgae, a strong effect of each spectral range on the lipid composition can be expected.

Light spectrum-dependent modification of lipid composition may be useful for the improvement of stress tolerance, which was tested in barley. The additional FR illumination markedly elevated the PG and PS levels of barley leaves after 10 days at 15 °C compared to the WL control, whereas the levels of other lipid classes remained unchanged [190]. At 5 °C, the additional FR illumination led to markedly increased levels of MGDG, PG, PE, phosphatidyl inositol (PI), and phosphatidyl serine (PS) already after 1 day. PE, PS, and PG are prominent lipid classes, which have important roles in signal transduction processes [191]. In addition, the elevated level of PG may promote the proper functioning of the thylakoid membrane at 5 °C [192]. The important alteration caused by FR treatment was the significant increment in the number of double bonds in the different molecular classes belonging to PE, which has been correlated with cold-acclimation in cereals [193,194,195,196]. However, their number should be kept under control since adverse environmental conditions may induce their peroxidation [197].

### 4.5. Regulation of Nucleic Acid Metabolism by Light Intensity and Spectrum

Nucleic acid metabolism is the most essential process in plant life. However, the light and redox regulation of the process, especially the role of light intensity and quality (Table A3), has received little attention in the past decade. One of the possible reasons for its under-researched status may lie in its fundamentality. For example, plants deficient in several enzymes of nucleotide metabolism show similar [198,199,200] or seedling-lethal phenotypes [201].

#### 4.5.1. Effect of Light Intensity on Nucleic Acid Metabolism

Many key enzymes of NTP and dNTP synthesis are affected by light intensity by the involvement of the redox system (Table A3) [202]. In rice (*Oryza sativa*), the ribonucleotide reductase (RNR) is required for chloroplast biogenesis in early leaf development since it affected the phenotype of OsRNR large and small subunit-deficient mutants, *v3* and *st1*, respectively, and it is light intensity-responsive [200]. The effect of light on RNR may be mediated by Trx based on its stimulating effect on RNR in *Arabidopsis* [203]. In addition, many other enzymes involved in nucleic acid metabolism are putative Trx targets in land plants, such as those ones participating in purine salvage, NTP and dNTP synthesis, pyrimidine de novo synthesis, and DNA remodelling (Table A3) [204].

During purine and pyrimidine salvage, nucleosides and nucleobases are recycled from metabolism or uptake into nucleotides. Proper salvage and/or degradation of nucleotides and related molecules may be essential during development [202] or under stress conditions and should be adjusted to the changes in the light conditions [205]. Accordingly, under extended darkness or increased leaf age, xanthine dehydrogenase (XDH, related to purine catabolism) is needed to delay senescence and to avoid excessive ROS accumulation of dark-stressed plants after re-exposition to light [205]. In addition, the expression of the *uracil phosphoribosyltransferase* (*UPRT*, related to uracil salvage) gene was found to be light-induced and peaked in the middle of the light period; furthermore, UPRT showed a pronounced function in the establishment of photosynthesis [206].

Not only nucleotide metabolism but also RNA synthesis and DNA repair processes exhibit light and redox dependency (Table A3). The presence of light can alter the nucleotide content of tRNAs [207]. Plastid-encoded RNA polymerase (PEP) is a principal RNA polymerase in chloroplasts and is affected by light-induced redox signals [201]. Proteins tightly associated with PEP core subunits, called polymerase-associated proteins (PAPs), are required for PEP activity. Most of the *PAPs* and *sigma factor* (which determines the promoter specificity of the major RNA polymerase) genes are light-induced. Furthermore, PEP and associated proteins (PEP complex) are major targets of photosynthesis-derived redox signals mediated by Trx-Z through the fructokinase-like proteins FLN1 and FLN2 and ECB1/MRL7 (EARLY CHLOROPLAST BIOGENESIS 2/MESOPHYLL-CELL RNAI LIBRARY LINE 7) in *Arabidopsis*. During light-dark transition, Trx-Z mediates redox signals to the PEP complex through FLN2. Fe-SOD2, Fe-SOD3, and PRDA1 (PEP-RELATED DEVELOPMENT ARRESTED 1) were reported to participate in the redox regulation of the PEP complex.

#### 4.5.2. Control of Nucleic Acid Metabolism by Light Spectrum

The influence of light spectrum on nucleotide metabolism was demonstrated in the case of thymidine kinase (TK), which transfers a phosphate group to a thymidine molecule to form TMP in the nucleotide salvage pathway [202]. Namely, BL downregulated the expression of the *TK* gene compared to RL [144]. In addition, nucleoside diphosphate kinases (NDPKs) showed specific phosphorylation after exposition to RL in pea (*Pisum sativum*) [208]. During the study of NDPK involvement in the light spectrum-dependent signalling, NDPK 2 was reported to act downstream of PhyA and PhyB in *Arabidopsis* leaves [209]. NDPKs also have a role in ROS response and redox signalling, as indicated by the interaction of NDPK1 with CAT in *Arabidopsis* [210]. Furthermore, NDPK1-overexpressing plants exhibited paraquat tolerance and enhanced elimination of exogenous H_2_O_2_ [210].

The control of RNA synthesis by light spectrum is indicated through the influence of phytochromes on the expression of multiple nuclear-encoded, chloroplast-targeted sigma factors, SIG1–6 [211]. The expression of *SIG2* and *SIG6* was highly upregulated under FRL or BL, compared to WL or RL. In *Arabidopsis*, SIG1-4 and SIG6 were strongly downregulated in *phy* mutants, whereas SIG5 showed less downregulation. However, SIG5 was shown to be mainly regulated by BL [211].

UV-induced DNA damage can be repaired via light and dark repair processes, of which BL is involved in light repair [212]. When damage is non-extensive, usually light-dependent damage repair is facilitated by photolyases, which absorb BL and revert DNA to its normal configuration. Dark repair, by contrast, is light-independent and tends to repair specific types of DNA damage. The repair of nucleic acids may also depend on the light dependency of the metabolism of their precursors, which was shown in rice and *Arabidopsis* [213,214].

### 4.6. Light Control of Secondary Metabolism

By definition, plant secondary metabolites have no direct functions in growth and development. However, they play a significant role in plant defence against pathogens or herbivores, attract pollinators or seed-dispersing animals, or serve as allelopathic agents, absorbers of UV radiation or defenders against oxidative stress. Based on chemical properties, these metabolites can be divided into three major groups: terpenes, phenolics, and nitrogen-containing compounds (e.g., alkaloids) [215]. The effect of different light intensities and wavelengths on the accumulation of various secondary metabolites has been studied in many plant species [216,217,218]. However, the light regulation of the metabolic pathways of these compounds in relation to plant redox status has not been discussed before. Since plant secondary metabolites include tens of thousands of compounds, we will focus only on key steps of their metabolism or on substances with significant importance in most plant species (Table A4). The light regulation of plant secondary metabolism in relation to redox processes has received more attention in the case of phenolics, especially anthocyanins, but less in other substances, like terpenes or alkaloids. The light control of the enzymes of terpenoid and flavonoid biosynthesis is mediated by HY5 targets (Table A4). HY5, in turn, is regulated by various photoreceptors, as described in the introduction [164].

#### 4.6.1. Effect of Light Intensity on Secondary Metabolism

Basically, terpenes (and their derivatives terpenoids, containing oxygen besides carbon and hydrogen) are the largest class of secondary products, and they are formed by the fusion of isoprene units [122]. Several enzymes of their synthesis are regulated by light conditions, the redox system, and the HY5 transcription factor (Table A4). Among terpenoids, quinones and chromanol have a role in the response to HL stress [219]. Light control of the biosynthesis of monoterpene precursors was shown in the Moth orchid (*Phalaenopsis bellina*) since the promoter of the gene encoding geranyl diphosphate synthase (GPPS, catalysing the mentioned process) was reported to have a light-responsive element [220]. However, it did not have HY5-interactive elements; therefore, its control is independent of this central regulator of light response. Similar to monoterpenes, the synthesis of triterpenes seems to be light-associated too, since the promoter of the *KcMS* gene, encoding a triterpene synthase, contains light-responsive elements in the mangrove plant *Kandelia candel* [221]. Light control of farnesyl diphosphate synthase (FPS), which is the key enzyme in isoprenoid (which consists of one or more isoprene units) biosynthesis, was shown in plants, too. The overexpression of its mitochondrial isoform (FPS1L) in *Arabidopsis*, for instance, caused leaf chlorosis under HL, which might be triggered by H_2_O_2_ accumulation [222]. Interestingly, shading enhanced FPS expression in *Aralia elata* (Miq.) Seem, in contrast to squalene synthase (SQS) and squalene epoxidase (SQE), two other enzymes of the isoprenoid metabolic pathway. However, more excessive shading downregulated the expression of all three enzymes [223]. The effect of light on the isoprenoid pathway may be connected to the redox system since the SQE-deficient *dry2/sqe1-5 Arabidopsis* mutant showed deficient ROS production and NADPH oxidase function [224].

Phenolics form a chemically diverse group of secondary metabolites with almost 10,000 individual substances, and one of their largest group consists of flavonoids [122]. The biosynthesis of flavonoids (flavones, flavonols, isoflavones, and anthocyanins) is affected by light and HY5 (Table A4) [225]. The connection of flavonoids with the redox system is based on their good ROS scavenging capacity [226]. However, there is little information available on the redox and light dependency of the biosynthesis of non-anthocyanin flavonoids. While flavonoid biosynthesis received more attention in the past 30 years, little is known about their catabolic processes, and the available information is mostly about anthocyanin degradation [227]. Enzymes catalysing the immediate formation of anthocyanins, flavones, and flavonols (UDP-sugar glucosyltransferase, UGT; anthocyanin acyltransferase, AAT; O-methyltransferase, OMT) are shared between the biosynthetic pathways of the three main types of these compounds [225]. *UGT* and *AAT* gene expression showed strong upregulation under HL treatment [228]. However, as was demonstrated in the work of [229], the changes in the activity of early or shared flavonoid biosynthetic enzymes might not result in a change in every flavonoid type compound [229]. The effect of light conditions on phenylalanine ammonia lyase (PAL), catalysing one of the earliest steps of flavonoid biosynthesis, may be mediated by the Trxs [50,204]. The connection between Trxs and flavonoid biosynthesis was proven in *Arabidopsis*, where the NADPH-dependent thioredoxin reductase (NTR) deficient double mutants *ntra ntrb* (NTRA: nuclear, NTRB: mitochondrial isoform) showed increased expression of the flavonoid biosynthesis-related transcription factor *MYB111/PFG3* (*MYB FAMILY TRANSCRIPTION FACTOR 111 /PRODUCTION OF FLAVONOL GLYCOSIDES 3*), the genes *F3′H/TT7* (*FLAVONOID 3′-HYDROXYLASE/TRANSPARENT TESTA 7 PROTEIN*), *F3H/TT6* (*FLAVANONE 3-HYDROXYLASE/TRANSPARENT TESTA 6 PROTEIN*), and *flavonol synthase 1 (FLS1)* [230]. A further relationship between flavonoids and the redox system was proposed by Akhtar et al. (2010) based on the induction of flavonoid biosynthesis by photosynthetic redox imbalance in *Lemna gibba* exposed to various stresses [231]. Since the relationship between the flavonoid synthesis and redox system was confirmed both in a dicot and monocot species, the light intensity-dependent redox changes seem to be general regulators of flavonoid levels in plants.

Anthocyanins are pigmented flavonoids, with sugars at position three or sometimes in another position [122]. Their HL-induced accumulation was demonstrated in *Arabidopsis*, and this change was redox-associated based on its fine-tuning by isoforms of TRX [232]. A further relationship of anthocyanins with the redox system is indicated by their antioxidative properties and role in the tolerance to abiotic stress-induced oxidative stress [233]. Under abiotic stress, the induction of anthocyanin biosynthesis on the upper epidermis of leaves is mediated by ROS signalling and transcription factors such as MYB/bHLH/WD40 in *Arabidopsis* [234,235]. The alterations in ROS formation may derive from the changes in the redox state of the photosynthetic electron transport chain which have been suggested to affect both AsA and anthocyanin accumulation [229]. In *Arabidopsis*, anthocyanin biosynthesis is regulated by H_2_O_2_ homeostasis, which is controlled by AsA and glutathione accumulation, and glutathione redox state under HL. Accordingly, the AsA-deficient mutant *vtc2-1* accumulated significantly less anthocyanin under HL [229]. This change may be the result of transcriptional regulation since in *vtc1* and *vtc2* mutants, the induction of the key transcription factors, PAP1 (PRODUCTION OF ANTHOCYANIN PIGMENT), GL3 (GLABRA 3), and EGL3 (ENHANCER OF GLABRA), as well as transcripts of anthocyanin biosynthesis enzymes, were HL-impaired. AsA deficiency may also affect anthocyanine levels through its influence on flavonoid biosynthesis since the activity of three related 2-oxoglutarate-dependent dioxygenases (2ODDs), F3H, FLS, and leucoanthocyanidin dioxygenase (LDOX), require AsA for the reduction of over-oxidized FeIV to FeII in the active site of the enzyme to avoid inactivation. In addition, low AsA content reduced the accumulation of phenylpropanoids, which play an important role in the biosynthesis of anthocyanins. Regarding the light-dependent, redox-mediated control of anthocyanin synthesis, it has also been shown that H_2_O_2_ or glutathione-mediated redox signals acted upstream of the PAP1 transcription factor [233]. Despite the enhanced anthocyanin biosynthesis capacity, the transgenic plants were, for instance, not able to induce anthocyanin synthesis under drought-induced oxidative stress. The possible mechanism of anthocyanin catabolism was suggested to be implemented via polyphenol oxidases (PPOs), anthocyanin β-glucosidase, or class III peroxidases (POXs) *in planta*, enzymes which link the process to the light-responsive redox system. PPOs are localised in the plastids, whereas POXs are distributed in several cell compartments, including vacuoles. Thus, POXs are more likely candidates for in vivo anthocyanin degradation [227,236]. Accumulation of POXs, phenolics, and AsA in the vacuoles and the apoplast is light-dependent, serving as an important sink for H_2_O_2_ [237]. A further link of the anthocyanin catabolism to the redox system is indicated by the observation that anthocyanin β-glucosidase and peroxidases could be Trx targets [204].

Alkaloids are a large, diverse group of nitrogen-containing secondary metabolites with more than 15,000 members. Most alkaloids participate in the defence against plant herbivores, especially mammals [122]. Because of their molecular diversity, in this paragraph, the light- and redox-associated biosynthetic responses of the most investigated alkaloid types, tropane and indole alkaloids, will be summarized, including their accumulation and the regulation of some early biosynthetic genes. However, it should be noted that the alteration of these early genes is not necessarily sufficient to enhance the final alkaloid yield [238]. Light acting through phytochromes has been suggested to modulate key enzymes involved in the metabolic pathways of alkaloids [215]. Indole alkaloid accumulation was reported to be induced by light and ROS, whereas tropane alkaloid production was diminished by light. The indole alkaloid metabolism in *Psychotria leiocarpa* (Cham. & Schltdl.) is linked to the redox system via N,β-D glucopyranosyl vincosamide (GPV), which is an N-glycosylated monoterpene indole alkaloid with ROS scavenging activity [239]. The GPV level is controlled by light since its accumulation was enhanced by darkness-to-light transition but diminished after light-to-darkness transfer. In addition, the nicotinic alkaloid synthesis catalysed by putrescine N-methyltransferase was also light- and ROS-sensitive in tobacco calli [240]. Both light and ROS negatively affected this reaction by downregulating PMT which catalyses the S-adenosyl methionine-dependent N-methylation of putrescine. The inhibition was mitigated by reducing H_2_O_2_ levels by CAT or 1,3-dimethyl-2-thiourea (DMTU, also a scavenger of O_2_^•−^ and HO^•^) application. Interestingly, DTMU treatment reduced alkaloid content in contrast to CAT or darkness, which might suggest a role of other ROS (O_2_^•−^ and/or HO^•^) in alkaloid biosynthesis of tobacco callus [240].

#### 4.6.2. Regulation of Secondary Metabolism by Light Spectrum

The effect of the light spectrum on secondary metabolism was not studied as intensively as that of the light intensity (Table A4). Considering the metabolism of terpenes, the regulation of FPS (isoprenoid synthesis) by light spectrum was shown in rice since BL enhanced the expression of the *FPS* gene [241]. This effect is probably mediated by Crys based on the enhanced *FPS* transcript abundance of transgenic *Artemisia annua* overexpressing the *AtCRY1* gene [242]. The metabolism of another group of terpenes, phytoenes, which are intermediates in the biosynthesis of carotenoids (tetraterpene derivatives containing oxygen), are synthesized from two molecules of geranylgeranyl pyrophosphate by phytoene synthase (PSY), was also affected by light spectrum [243]. PSY activity was enhanced by RL and WL, but not by FRL compared to darkness during mustard photomorphogenesis [243]. Interestingly, PSY protein content was correlated negatively with PSY activity which indicates a negative feedback regulation [243]. The phytochrome-related control of the *PSY* gene was demonstrated in *Arabidopsis* since PIF1 and PIF3 repressed it under RL illumination [244].

The biosynthesis of flavonoids is generally activated under UV exposure [225]. Besides UV, BL induced FLS1, which catalyses the oxidation of flavanones to flavones, in parsley [245]. Interestingly, the effect of UV-B on the level of a flavonoid, quercetin 3-galactoside was differently modified by RL and FRL in silver birch (*Betula pendula* Roth) since it was higher after UV-B + RL treatment than after UV-B + FRL treatment [246]. In addition, the amount of two other phenolics, chlorogenic acid and a cinnamic acid derivative, was influenced by the FRL: RL ratio in this species. These observations indicate that the ratio of the various spectral ranges is important in the control of the metabolism of phenolics.

The control of anthocyanin synthesis by light spectrum was shown in apple (*Malus domestica*) peel, where anthocyanin synthesis was increased by the UV range of sunlight through upregulation of the MdMYB10 transcription factor and key enzymes of anthocyanin biosynthesis: PAL, chalcone synthase (CHS), flavanone-3-hydroxylase (F3H), dihydroflavonol 4-reductase (DFR), and anthocyanidin synthase (ANS) [247]. Lettuce plants grown under a 3:1 ratio of RL to BL accumulated more anthocyanin, which was derived from the transcriptional regulation of the related biosynthesis genes [248].

The level of indole alkaloids was also influenced by the light spectrum since RL increased the level of two of them, vindoline and catharanthine, in Madagascar periwinkle (*Catharanthus roseus* L.) [249]. This RL control is mediated by the photoreceptor-responsive HY5 transcription factor, which can also coordinate the process with other metabolic pathways and the redox regulation through its corresponding target genes [250]. In addition, the amount of GPV was increased by red, blue, or FR monochromic lights in photomorphogenic *P. leiocarpa* plants [239]. Although there are some studies available discussing the effect of various spectral ranges on the accumulation of alkaloids and the other discussed secondary metabolites, the underlying molecular regulatory mechanisms need further investigation.

## 5. Conclusions

Light intensity and spectral composition play a major role in the adjustment of primary and secondary metabolism to changing environmental conditions in plants. A successful metabolic adjustment is responsible for the optimization of the growth and development of plants and can reduce the damage caused by unfavourable environmental conditions. The effect of light on metabolic pathways is mediated by the various photoreceptors and/or the redox system, ensuring sufficient availability of reductants and oxidants for the various biochemical reactions, as summarised for carbon metabolism, nitrate, and sulphate assimilation in Figure 5. The light- and/or redox-responsive transcription factors such as HY5 play a major role in this metabolic regulation. The photosynthetic and mitochondrial electron transport chains are also important participants in this adaptation process since they are the major sources of ROS, affecting the redox environment in cells and tissues. The two main regulatory pathways of the light condition-dependent control of metabolism may interact with each other through HY5 or their other components.

Differences in the redox environment of the subcellular compartments strongly affect metabolic processes. Redox-mediated effects on the light intensity- and spectrum-dependent alterations of the primary and secondary metabolism in the individual cell compartment should be clarified, providing important future research directions.

## Abbreviations

^1^O_2_	singlet oxygen
(Fd/Trx)	ferredoxin/thioredoxin
2-OGDH	2-oxoglutarate dehydrogenase
ABA	abscisic acid
ADP	adenosine diphosphate
ANS	anthocyanidin synthase
APX	ascorbate peroxidase
AsA	ascorbic acid
ATP	adenosine triphosphate
BL	blue light
CAT	catalase
CBC	Calvin–Benson cycle
CHS	chalcone synthase
COP1	CONSTITUTIVELY PHOTOMORPHOGENIC1
Cry	cryptochromes
*CS*	citrate synthase
DFR	dihydroflavonol-4-reductase
DGD	digalactosyl-diacylglycerol
DMTMU	1,3-dimethyl-2-thiourea
E_GSH_	glutathione redox potential
F3H	flavanone-3-hydroxylase
FBP	fructose-1,6-bisphosphatase
FRL	far-red light
FUM	fumarase
γ-EC	γ-glutamylcysteine
γ-ECS	γ-glutamylcysteine synthetase
GAPDH	glyceraldehyde-3-phosphate dehydrogenase
GL	green light
GR	glutathione reductase
Grxs	glutaredoxins
GSH	reduced glutathione
GSSG	oxidized glutathione (= glutathione disulfide)
GSSG/GSH	ratio of oxidized and reduced glutathione
GSTs	glutathione S-transferases
H_2_O_2_	hydrogen peroxide
HL	high light intensity = high PPFD
HO^•^	hydroxyl radical
Hy5	ELONGATED HYPOCOTYL5
IDH	NAD-dependent isocitrate dehydrogenase
LL	low light intensity = low PPFD
MDHR3	monodehydroascorbate reductase 3
MGDG	monogalactosyl-diacylglycerol
NADP	nicotinamide adenine dinucleotide phosphate
NDPKs	diphosphate kinases
NRT2.1	high-affinity nitrate transporter
O_2_^•−^	superoxide radical
PAL	phenylalanine ammonia lyase
PAR	photosynthetically active radiation
PCD	programmed cell death
PDC	pyruvate dehydrogenase complex
PFT1	PHYTOCHROME AND FLOWERING TIME 1
PG	phosphatidylglycerol
PHOT	phototropins
Phy	phytochromes
PhyA	phytochrome A
PhyB	phytochrome B
PI	phosphatidylinositol
PMT	putrescine N-methyltransferase
PODs	peroxidases
PPFD	photosynthetic photon flux density
PRXs	peroxiredoxins
PS	phosphatidylserine
PSY	phytoene synthase
PUFAs	polyunsaturated fatty acids
R:FR	red/far-red ratio
RL	red light
RNR	ribonucleotide reductase
ROS	reactive oxygen species
RPK	ribulose-5-phosphate kinase
SA	salicylic acid
SAA	systemic acquired acclimation
SAS	shade avoidance syndrome
SBP	sedoheptulose-1,7-bisphosphatase
SOD	superoxide dismutase
SQE	squalene epoxidase
SQS	squalene synthase
TCA	tricarboxylic acid
TK	thymidine kinase
TRXs	thioredoxins
UV-B	ultraviolet-B light
UVR8	UV RESISTANCE LOCUS 8
WL	white light

## Appendix A

**Table A1:** Light- and/or redox-associated regulation of key enzymes or metabolites of the important metabolic processes.

		Light Regulation		HY5 Associated Regulation	Redox (Light Related) Associated Regulation	High Light		Light Spectra Specific Regulation					
								Blue Light		Red Light		Far-Red Light	
Metabolic Process		Enhancing Effect	Diminishing Effect or Dark			Enhancing Effect	Diminishing Effect	Enhancing Effect	Diminishing Effect	Enhancing Effect	Diminishing Effect	Enhancing Effect	Diminishing Effect
**Carbohydrate metabolism**	**Anabolism**	Rubisco, GAPDH, FBP, SBP, RPK [122]	N.D.	Rubisco, GAPDH, FBP, SBP, RPK [123]	Fd/Trx system: GAPDH, FBP, SBP, RPK [128,129]; H_2_O_2_: CBC enzymes [131]	GPT [131]	N.D.	Rubisco [27,140,141]	N.D.	N.D.	Rubisco [27,140,141]	Low R:FR: Rubisco [127]	N.D.
	**Catabolism**	FUM, MDH2 [137]	PDC, CS, ACO, IDH [137] PFK [134]	PDC, ACO, FUM, NAD-ME [123]	H_2_O_2_, glutathion, Trxs, Grxs: GAPC [183]; Fd/Trx pathway, TrxL2 pathway: PFK [134]; Trxs: TCA cycle enzymes [35,136,185]	TCA cycle [136]	N.D.	N.D.	2-OGDH [146]	N.D.	2-OGDH [146]	2-OGDH [146]	N.D.
**Nitrogen assimilation**		NR [148]	N.D.	NRT2.1, nitrate uptake [124,125,126,127]; NR [7,97,150,151]	NAD(P)H/NAD(P)+ [152]; GSH/GSSG [67]	N.D.	N.D.	N.D.	NR (WL+FRL) [67]	N.D.	N.D.	N.D.	NR (WL+FRL) [67]
**Amino acid levels**		Amino acids, except for: Pro, Met, Thr [67]	N.D.	N.D.	Amino acid content [154]	N.D.	N.D.	Amino acids (WL+BL) [91], [159]	N.D.	N.D.	N.D.	Glu, Pro, Gly, Thr etc. (low R:FR) [161]	N.D.
**Sulphur assimilation**		APR [190]; Sulphate reduction [67]	N.D.	APR [205,206]; AtSULTR1;2 [164]	N.D.	N.D.	N.D.	N.D.	Sulphate reduction (WL+BL) [67]	N.D.	N.D.	N.D.	Sulphate reduction (WL+ FRL) [67]
**Glutathione metabolism**		N.D.	N.D.	GST [123]	GSH levels [154,170]	GS [67]; GR, APX [152]	N.D.	N.D.	GS (WL+BL) [67]	GSTs [72,167]	N.D.	N.D.	GSH, GS, GR (WL+ FRL) [67];

GPT: glucose-6-phosphate/phosphate translocator; CBC: Calvin-Benson Cycle; TCA: tricarboxylic acid; Rubisco: ribulose-1,5-bisphosphate carboxylase oxygenase; GAPDH: glyceraldehyde-3-phosphate dehydrogenase; FBP: fructose-1,6-bisphosphatase; Fd: ferredoxin; SBP: sedoheptulose-1,7-bisphosphatase; RPK: ribulose-5-phosphate kinase; Trx: thioredoxin; HY5: elongated hypocotyl 5; HL: high light; GAPC: cytosolic GAPDH; GAPC; Grx: glutaredoxine; PFK: phosphofructokinase; TrxL2: Trx-like2; MDH: malate dehydrogenase; NAD-ME: NAD-dependent malic enzyme; FUM: fumarase; CS: citrate synthase; IDH: isocitrate dehydrogenase; ACO: aconitase; PDC: pyruvate dehydrogenase complex; 2-OGDH: 2-oxoglutarate dehydrogenase; GR: glutathione reductase; GST: glutathione S-transferase; NR: nitrate reductase; APX: ascorbate peroxidase; GSSG: glutathione disulphide; GSH: reduced glutathione; GS: glutathione synthase; APR: phosphosulphate reductase; SULTR1;2:Sulphate transporter1and 2; WL: white light, BL: blue light; FRL: far-red light; R:FR: red: far-red ratio; NRT2.1: High-affinity nitrate transporter N.D.: no available data.

**Table A2:** Light and/or redox associated regulation of certain enzymes (or genes) involved in lipid biosynthesis.

Enzyme	Light-Associated	Redox Associated	Light Intensity Dependence	Spectral Dependency	HY5 Target
FAD2	Yes [176]	N.D.	N.D.	N.D.	N.D.
FAD3	Yes [177]	N.D.	N.D.	N.D.	Yes [123]
FAD4	N.D.	Yes (reviewed by [174])	N.D.	N.D.	N.D.
FAD6	N.D.	Yes (reviewed by [174])	N.D.	N.D.	N.D.
FAD7	Yes [175]	N.D.	N.D.	N.D.	N.D.
FAD8	Yes [177]	N.D.	N.D.	N.D.	Yes [123]
ACCase	Yes (reviewed by [174])	Yes (reviewed by [174])	Yes (reviewed by [174])	N.D.	N.D.
MGD	N.D.	Yes (reviewed by [174])	N.D.	N.D.	N.D.
SAD	N.D.	Yes (reviewed by [174])	Yes [55]	N.D.	Yes, putative [123]
DGD1	N.D.	N.D.	N.D.	N.D.	Yes [123]
PDAT	Yes [178]	N.D.	N.D.	N.D.	N.D.
MCAT	N.D.	Yes [173]	N.D.	N.D.	N.D.
PI4K	Yes [180]	N.D.	N.D.	Yes [180]	N.D.
FAS	Yes [186]	N.D.	N.D.	Yes [186]	N.D.

FAD4: phosphatidylglycerol desaturase; FAD6: chloroplast omega-6 desaturase; FAD7: chloroplast omega-3 desaturase; FAD8: chloroplast omega-3 desaturase; FAD 2: omega 6 fatty acid desaturase; FAD3: endoplasmic reticulum omega-3 desaturase; MGD: MGDG synthase; SAD: stearoyl-ACP desaturase; FAD 2: omega 6 fatty acid desaturase; DGD1: digalactosyldiacylglycerol synthase 1; PDAT: phospholipid:diacylglycerol acyltransferase; PI4K: phosphatidylinositol 4-kinase; MCAT: Malonyl CoA:ACP transacylase; FAS: fatty acid synthase; N.D.: no available data..

**Table A3:** Light- and/or redox-associated regulation of proteins involved in nucleotide or nucleic acid metabolism.

Enzyme/Protein	Light-Associated	Redox-Associated	Spectral Dependency	Light Intensity Dependence
**Purine and pyrimidine nucleotide de novo synthesis**				
DHODH	N.D.	Yes, putative (reviewed by [204])	N.D.	N.D.
**NTP and dNTP synthesis**				
RNR	Yes [200,203]	Yes [203]	N.D.	Yes [200]
AMK	N.D.	Yes (reviewed by [204])	N.D.	N.D.
NDPK	Yes [209]	Yes (reviewed by [204])	Yes [209]	N.D.
TK	Yes [144]	N.D.	Yes [144]	N.D.
UMK	Yes [213]	N.D.	N.D.	N.D.
**Purine salvage**				
ADK	N.D.	Yes (reviewed by [204])	N.D.	N.D.
XDH	Yes [205]	Yes [205]	N.D.	N.D.
**Pyrimidine salvage**				
UPRT	Yes [199,206]	N.D.	N.D.	N.D.
UKL1 and 2	Yes [214]	N.D.	N.D.	N.D.
**mRNA synthesis**				
PEP complex	reviewed by [201]	reviewed by [201]	N.D.	N.D.
SIG2; SIG5; SIG6	Yes [211]	N.D.	Yes [211]	N.D.
SIG1, SIG3-4	Yes [211]	N.D.	N.D.	N.D.
**DNA remodelling**				
ATP-dependent DNA helicase	N.D.	Yes (reviewed by [204])	N.D.	N.D.
**DNA repair**				
Photolyases	Yes (reviewed by [212])	N.D.	Yes (reviewed by [212])	N.D.

DHODH: dihydroorotate dehydrogenase; RNR: ribonucleotide reductase; AMK: adenylate kinase; NDPK: nucleoside diphosphate kinase; TK: thymidine kinase; UMK: UMP kinase; ADK: adenosine kinase; XDH: xanthine dehydrogenase; UPRT: uracil phosphoribosyltransferase; UKL: Uridine Kinase Like; PEP: plastid-encoded RNA polymerase; SIG: sigma factor; N.D.: no available data.

**Table A4:** Light and/or redox associated regulation of certain proteins (or genes) involved in secondary metabolism.

Enzyme/Pathway	Light-Associated	Redox-Associated	Light Intensity Dependence	Spectral Dependency	HY5 Target
**Terpenoid biosynthesis**					
GPPS	Yes [220]	N.D.	N.D.	N.D.	No [220] YES, putative [123]
FPS	Yes [241,242]	N.D.	Yes [224]	Yes [241]	N.D.
FPS1L	N.D.	N.D.	Yes [222]	N.D.	N.D.
SQS	N.D.	N.D.	Yes [223]	Yes [223]	Yes [123]
SQE	N.D.	N.D.	Yes [223]	Yes [223,243,244]	Yes [123]
PSY	Yes [243]	N.D.	N.D.		Yes [123]
KcMS	Yes [221]	N.D.	N.D.	N.D.	N.D.
**Earlier flavonoid biosynthesis and non-anthocyanin flavonoid biosynthesis**					
UGT	N.D.		Yes [228]	N.D.	Yes [123]
AAT	N.D.		Yes [228]	N.D.	N.D.
PAL	N.D.	Yes [204,247]	N.D.	N.D.	Yes [123]
CHS	N.D.	Yes [247]	N.D.	N.D.	Yes [123]
MYB111/PFG3 TF	N.D.	Yes [230]	N.D.	N.D.	N.D.
F3′H/TT7	N.D.	Yes [230]	N.D.	N.D.	Yes [123]
F3H/TT6	N.D.	Yes [230]	N.D.	N.D.	Yes [123]
FLS1	N.D.	Yes [230]	N.D.	Yes [245]	Yes [123]
**Anthocyanin biosynthesis**					
MYB/bHLH/WD40 TF	N.D.	Yes [234,235]	N.D.	N.D.	N.D.
MYB10 TF	N.D.	Yes [247]	N.D.	N.D.	N.D.
ANS	N.D.	Yes [247]	N.D.	N.D.	N.D.
F3H	N.D.	Yes [247]	N.D.	N.D.	Yes [123]
DFR	N.D.	Yes [247]	N.D.	N.D.	Yes [123]
PAP1	N.D.	Yes [229]	N.D.	N.D.	N.D.
GL3	N.D.	Yes [229]	N.D.	N.D.	N.D.
EGL3	N.D.	Yes [229]	N.D.	N.D.	N.D.
LDOX	N.D.	Yes [229]	N.D.	N.D.	Yes [123]
**Anthocyanin catabolism**					
β-glucosidase	N.D.	Yes [204]	N.D.	N.D.	N.D.
POXs	Yes [237]	Yes [204]	N.D.	N.D.	N.D.
**Tropane and indole alkaloid biosynthesis ***					
PMT	Yes [240]	Yes [240]	N.D.	N.D.	N.D.

GPPS: geranyl diphosphate synthase; FPS: farnesyl diphosphate synthase; FPS1L: FPS mitochondrial isoform; MYB111/PFG3: MYB FAMILY TRANSCRIPTION FACTOR /PRODUCTION OF FLAVONOL GLYCOSIDES 3; SQS: squalene synthase; SQE: squalene epoxidase; PSY: phytoene synthase; KcMS: *Kandelia candel* multifunctional triterpene synthase; PAL: Phenylalanine ammonia lyase; AAT: anthocyanin acyltransferase; UGT: UDP-sugar glucosyltransferase; CHS: chalcone synthase; F3′H/TT7: FLAVONOID 3′-HYDROXYLASE/TRANSPARENT TESTA 7 PROTEIN, F3H/TT6: Flavanone 3-hydroxylase/transparent testa 6 protein; FLS1: Flavonol synthase 1; ANS: anthocyanidin synthase; F3H: flavanone-3-hydroxylase; DFR: dihydroflavonol 4-reductase; PAP1: production of anthocyanin pigment; GL3: glabra 3; EGL3: enhancer of glabra; LDOX: leucoanthocyanidin dioxygenase; POXs: class III peroxidases; PMT: Putrescine N-methyltransferase; N.D.: no available data.* both tropane and indole alkaloids showed spectrum-dependent accumulation, however, the light and/or redox regulation of main biosynthetic enzymes were not investigated yet.
